# Delivering Pediatric Palliative Care: From Denial, Palliphobia, Pallilalia to Palliactive

**DOI:** 10.3390/children5090120

**Published:** 2018-08-31

**Authors:** Stefan J. Friedrichsdorf, Eduardo Bruera

**Affiliations:** 1 Children’s Hospitals and Clinics of Minnesota, 2525 Chicago Ave S, Minneapolis, MN 55403, USA; 2 University of Minnesota Medical School, 420 Delaware Street SE, Minneapolis, MN 55455, USA; 3 Department of Palliative Care and Rehabilitation Medicine, The University of Texas, MD Anderson Cancer Center, 1515 Holcombe Blvd., Houston, TX 77030, USA; ebruera@mdanderson.org

**Keywords:** pediatric palliative care, program development, barriers, hospice, myths, program implementation

## Abstract

Among the over 21 million children with life-limiting conditions worldwide that would benefit annually from a pediatric palliative care (PPC) approach, more than eight million would need specialized PPC services. In the United States alone, more than 42,000 children die every year, half of them infants younger than one year. Advanced interdisciplinary pediatric palliative care for children with serious illnesses is now an expected standard of pediatric medicine. Unfortunately, in many institutions there remain significant barriers to achieving optimal care related to lack of formal education, reimbursement issues, the emotional impact of caring for a dying child, and most importantly, the lack of interdisciplinary PPC teams with sufficient staffing and funding. Data reveals the majority of distressing symptoms in children with serious illness (such as pain, dyspnea and nausea/vomiting) were not addressed during their end-of-life period, and when treated, therapy was commonly ineffective. Whenever possible, treatment should focus on continued efforts to control the underlying illness. At the same time, children and their families should have access to interdisciplinary care aimed at promoting optimal physical, psychological and spiritual wellbeing. Persistent myths and misconceptions have led to inadequate symptom control in children with life-limiting diseases. Pediatric Palliative Care advocates the provision of comfort care, pain, and symptom management concurrently with disease-directed treatments. Families no longer have to opt for one over the other. They can pursue both, and include integrative care to maximize the child’s quality of life. Since most of the sickest children with serious illness are being taken care of in a hospital, every children’s hospital is now expected to offer an interdisciplinary palliative care service as the standard of care. This article addresses common myths and misconceptions which may pose clinical obstacles to effective PPC delivery and discusses the four typical stages of pediatric palliative care program implementation.

## 1. Introduction

The special edition “Pediatric Palliative Care” in *Children* (http://www.mdpi.com/journal/children/special_issues/palliative_care) has collated 20 outstanding articles from many of the leading pediatric palliative care researchers and clinicians worldwide allowing us to present an overview of advances, research, and challenges in pediatric palliative care (PPC). As the guest editor, I thank the authors for their strong contributions to this edition, in assisting children with serious illness and their families, as well as to moving our field further along.

Over 21 million children 0–19 years would benefit annually from a palliative care approach worldwide, more than eight million needing specialized PPC [1,2]. In the United States alone, more than 42,000 children died in 2013, fifty-five percent of them infants younger than one year [3]. The leading causes of pediatric deaths include accidents (7645 children), suicide (2143), and homicide (2021). Leading life-limiting conditions include congenital malformations and chromosomal abnormalities (5740) followed by malignancies (1850). Minorities, such as Latinos, appear to have higher barriers to accessing PPC [4].

PPC is about matching treatment to patient goals and is considered specialized medical care for children with serious illness. It is focused on relieving pain, distressing symptoms, and stress of a serious illness and appropriate at any age and at any stage, together with curative treatment. The primary goal is to improve quality of life for both the child and his or her family. In the words of an ill child: “Palliative care no longer means helping children die well, it means helping children and their families to live well, and then, when the time is certain, to help them die gently.” (Mattie Stepanek, 1990–2007). Sadly, advances in the control of symptoms in children dying of diseases such as cancer have not kept pace with treatment directed at curing the underlying disease. The majority of distressing symptoms in children with advanced cancer (such as pain, dyspnea and nausea/vomiting) were not treated, and when treated, therapy was commonly ineffective [5,6,7,8,9].

In this editorial we will address the four steps of PPC program implementation [10], followed by an evaluation of common assumptions, myths and barriers, which may hinder the implementation of PPC into the care of a child or teenager with serious illnesses [11,12].

## 2. Implementing Institutional Change: The Four Stages of PPC Program Implementation

Although the majority of children’s hospitals in the United States do have a pediatric palliative care program, most of them appear to be understaffed and underfunded. A survey by Feudtner et al. in 2013 among 226 US children’s hospitals (of which 162 responded) showed that 112 (69% of respondents; at least 49.6% of all children’s hospitals) have PPC program [13]. However, most programs offer only inpatient services, and most only during the work week.

A universal reality is that, overwhelmingly, in places where palliative care has not existed before, will require major cultural adaptation [10]. We have adapted the following four stages, initially described by Bruera in 2004 [10], to pediatric palliative care program implementation.

### 2.1. Stage 1: Denial

Clinical leaders suggesting the implementation of a new PPC program in a pediatric institution are very likely to face significant denial by clinical colleagues and hospital executives, who may not be aware of the need for a PPC program. Often, there are limited or no measurement of the amount of physical and emotional distress suffered by children and their families with serious illness. The limited documentation on the need of PPC is complicated by perceptions (which are unfortunately not based on reality) such as “Our patients here usually have very good symptom control” or “We here at…” (insert the name of any pediatric primary service or subspecialty here) “…already got it and we cover everything and don’t need an extra palliative care service.”

An important approach to expect and address denial, would be to carefully and rapidly document the level of unmet need in patients and families in the pediatric institution, including in the patients under care by individuals, who are in denial. Simple surveys of uncontrolled symptoms or emotional distress might be extremely useful. Parents’ testaments, in writing or in a short movie clip, might be particularly helpful to overcome denial during a presentation to colleagues and leadership.

### 2.2. Stage 2: Palliphobia

The second stage of PPC program implementation in a pediatric institution can be best described as the recognition that there is a problem, but usually this meets consistent fear about consequences of the problem and the possible solutions it entails. Although this might represent fear of the unknown, it is not unheard of that sometimes people react with great anger towards it. In fact, many individuals can react in negative way to developing a PPC team. Physicians, nurse practitioners and other health professionals may feel their professional competence is being questioned or even threatened by the new PPC team. In fact, they may be afraid of referring patients to the PPC service because of concerns over criticism of their symptom control, communication or overall treatment strategies they have used for many years. Hospitals executives may be afraid of being found lacking in compassion and holistic care among all the competing priorities in a shifting landscape of pediatric healthcare and scarce funding.

Common phrases PPC teams may hear include “The parents are not ready”, “We asked them and they said no” (although surprisingly few parents are asked for permission to include other services, such as infectious disease services),“She is not dying now”, “There is always something else we can try”, “It is too early”, “Hospice? That’s wonderful, but that is for other people”, “You talking to the family means destroying hope”, “We are still fighting”.

Unfortunately, further exacerbation of Palliphobia can be expected, if the PPC team tries too hard and/or is too fast in making changes in patient care. After a major confrontation with the neonatologist, oncologist etc. (a.k.a. the bull-in-the-china-shop-strategy—“move aside, the palliative care doctor has arrived…”), the PPC usually succeeds in changing and improving analgesia, communication strategies or changing discharge planning, but likely will not receive further consults/referrals from this team or unit, resulting in a negative reputation among colleagues in the institution.

A useful technique is to approach a limited number of possible referring clinicians and ask them to become supporters and mentors of the program. Once a significant number of patients have been treated, it is then safe to make a presentation in Grand rounds or Medical staff meetings showing the results, ideally with the presence of the referring clinicians. In absence of data, all opinions (including palliphobic ones) are good, but the presence of data on positive outcomes and testimonials from the initial team of referring clinicians can be very reassuring to those who are still uncommitted to refer patients.

“Palliphobia” is more difficult to overcome than “Denial”, and requires disciplined planning and rapid conflict resolution: Useful strategies to overcome this stage may include making great efforts at reassuring the existing clinical pediatric team that the PPC of course will work in an integrated fashion collaboratively with them. Importantly, members of the PPC team will not disqualify their patient care plan, but rather enhance them by focusing on aspects not addressed so far. “How can I help?” is commonly a useful question posed to the primary clinician, followed by a detailed discussion of who does what (“Would you like me to prescribe the methadone, or do you prefer me simply giving recommendations and you take care of it?”).

### 2.3. Stage 3: Pallilalia

A large number of PPC programs worldwide appear to be stuck in the 3rd stage “Pallilalia”. Usually about two to four years after implementing a PPC program, repetitive absurdities are spoken about palliative care in general and the PPC program specifically, without anything being done to advance its development. This is in fact the most dangerous stage, as it has a high risk resulting in burnout among PPC professionals.

Hospital leadership and executives describe PPC as “very important” or “a major priority”, but actually there’s no significant allocation of personnel, space, money, curriculum time etc. Colleagues within the pediatric institution frequently talk about how useful they feel PPC is, and how happy they are with the PPC team, but as a matter of fact refer only small minority of their patients—usually the ones with terrible psycho-social-medical and/or mental health problems. With this attitude, a PPC program is simply financially and administratively not viable.

Children’s hospitals at this stage frequently “appoint a committee to discuss PPC”, or propose “a major study whether PPC works in our institution” or, suggest the PPC lead applies for external grants, so “funds can be obtained for a pilot program in a year or two”. There have also been cases, where hospital leadership decided that the 6-figure donation to the PPC program were used to offset general losses in other departments instead.

Most understaffed PPC programs (commonly lead by a 0.2 FTE physician, who was on-call 24/7 and during his/her vacations), have disappeared at this stage. Clinical colleagues and institutions became used to the beneficial presence of the PPC team, while having made no major commitment to support it. 

A useful technique to overcome Pallilalia is to use benchmarks for clinical and time burden that might allow for fair comparisons. While some of the referring teams may see a mix of more and less time demanding encounters, palliative care teams always see time demanding patients and they frequently allow the referring teams to shorten their own encounter with the patients they refer. Therefore, clinical time may be a better measure than number of encounters. Since this is not always easy to measure, one palliative care team compared data from parking records to determine the additional burden on the palliative care team.

It is important to anticipate this developmental stage by gathering data of provided clinical services, number of patients seen, details of teaching conducted, research studies, etc. The PPC team should aim mostly at leaders of hospital and medical schools, rather than just immediate supervisors or peers, when providing documentation of work. They should request, that information be compared to output and resources of other programs within the institution and on a national level. PPC teams may ask for external review by national or international PPC leaders in the field. Sadly, not infrequently, only through resignation of the PPC leader, institutions come to realize that PPC has been badly under-resourced, and start corrective measures during the process of recruiting a successor.

### 2.4. Stage 4: Palliactive

A PPC program has reached the final level of development, when recognized by robust appointment and funding of professional interdisciplinary team PPC members. It is recommended to seek a designation of an administrative structure equal to oncology or infectious disease programs (department or division). Other important components of this stage include allocation of space, formal curriculum space and medical training program rotations.

PPC is truly recognized when colleagues actively refer patients to PPC and encourage other colleagues to do the same. Unfortunately, regression can happen at any time—frequently due to changes in administrative leadership within the institution. Also, in large children’s hospitals on certain units is not unlikely that a robust established PPC team may encounter all four stages on the very same day, depending on which physician they encounter.

## 3. Common Myths and Misconceptions in Pediatric Palliative Care (PPC)

### 3.1. Myth 1: PPC Is Primarily for Children with a Malignancy 

The majority of children dying of serious illness do not have cancer [14]. In 2013 a total of 42,328 children 0–19 years died in the United States, more than 55% (23,440) of them infants younger than one year [3]. The leading causes of pediatric deaths include accidents (7645 children), suicide (2143), and homicide (2021). In 2015 a total of 11,933 children, adolescents and young adults 0–24 years died due to a life-limiting disease: Leading conditions include congenital malformations and chromosomal abnormalities (5965), followed by malignancies (2688), and heart disease (1354) [15]. Although the prognosis for children with cancer has improved considerably over the last decades, malignancies now remains the leading cause of non-accidental death in childhood only in children older than one year of age. In pediatric cancer units, the presence of “trigger diagnoses” (triggering automatic referral to PPC) increased likelihood of palliative principle introduction 3.4 times (*p* < 0.003) [16].

That said, when providing interdisciplinary PPC services for children, it can be expected that most children do not have a malignancy [17] and about half of the patients would be infants. Despite the great need in neonatology, more than 45% of institution in Canada and the United States not have neonatal comfort care guidelines, and of those reporting institutional neonatal comfort care guidelines, 19.1% do not address pain symptom management [18]. More than 90% of respondents in the same study felt that their institution would benefit from further education and training in neonatal EOL care. Carter elaborates on PPC for babies in his article [19] in this special edition.

### 3.2. Myth 2: PPC Begins when Curative Treatments Stop 

Sometimes clinicians, patients and families incorrectly assume that PPC is only appropriate when all curative treatments are exhausted and discontinued and/or when a child is close to dying. In fact, PPC is recommended to commence at diagnosis of a life-threatening disease, to continue through the trajectory of the illness, and does not equal end-of-life care (but certainly includes it). PPC services extend beyond the child’s death to the family during bereavement [20]. Earlier recognition by both physicians and parents that the child had no realistic chance of cure led to a stronger emphasis on treatment to lessen suffering and integrate PPC in pediatric cancer patients [21].

The overall improvement in the prognosis of serious illnesses, and the emotional involvement in trying to save the life of a child may prevent both physicians and parents from discontinuing therapies. The pursuit of such therapy modalities may overshadow attention to advanced prevention and control of distressing symptoms and to quality of life and, which unfortunately may result in increased suffering during child’s end-of-life period. However, it is sometimes not possible for parents and/or the child to forgo further disease-directed treatment, and this should not be required in order to achieve optimal palliative care. The need to ensure that everything possible has been done may be the only way that some parents can live and cope with their child’s death [22]. 

The 2010 “Concurrent Care for Children” requirement of the United States Affordable Care Act has this at heart: The 2016 briefing of the Mary J. Labyak Institute for Innovation at the National Center for Care at the End of Life [23] described concurrent care for children with serious illness as follows: “Until 2010, parents in all but a few states in the United States were faced with forgoing curative treatments for their children to be eligible for hospice services. Or conversely, they were not eligible for beneficial interdisciplinary hospice services while getting curative treatment. The Patient Protection and Affordable Care Act (ACA) changed that situation. It requires all state Medicaid programs to pay for both curative and hospice services for children under age 21 who qualify. On 23 March 2010, President Obama signed ACA into law enacting a new provision, Section 2302, termed the “Concurrent Care for Children” Requirement (CCCR). Section 2302 states that a child who is eligible for and receives hospice care must also have all other services provided, or have payment made for, services that are related to the treatment of the child’s condition.1 This provision affects children who are eligible for Medicaid or the Children’s Health Insurance Program (CHIP). In its simplest form, implementation of this provision could be accomplished by the state Medicaid agency eliminating any provider claims that deny or delay concurrent curative care and hospice claims [23].” 

Pediatric oncology providers in a recent survey [24] issued a highly favorable opinion about their institution’s PPC service and agreed that early consultation is ideal. However, they report formally consulting PPC is extremely difficult because of what the PPC symbolizes to families and the emotional labor that the oncology provider must manage in introducing them.

### 3.3. Myth 3: Pediatric Palliative Care Involvement Shortens Life

Clinicians trained in palliative care will never issue a statement such as “There is nothing else we can do”. Quite the opposite, advanced PPC teams may say “there is always a lot we can do”. Even when the underlying life-limiting disease cannot be cured, sophisticated medical technology will be utilized to improve the quality of life of the child and his and her family and to prevent and treat distressing symptom. As such, PPC is therefore a very active and advanced approach to symptom management and family support.

It appears, that a palliative care consult for patients with serious illness is associated with longer survival and better quality of life: In the ENABLE III study, patients who received early palliative oncology care had significantly longer 1-year survival rates than those who received delayed palliative care [25]. In another innovative randomized controlled trial adults with advanced lung cancer receiving a palliative care intervention (providing appropriate and beneficial treatments) at the point of diagnosis, in fact increased the quality of life, decreased depression, and led to a prolonged life (11.6 months vs. 8.9 months) [26]. These results underscore the need for palliative care early in a serious illness and refute the notion that palliative care means giving up. Patients received palliative care alongside their curative treatment. There is now emerging evidence that the inclusion of PPC specialists improves the outcome of children with advanced serious illnesses, and sometimes represents the means to allows for curative care through advanced symptom management provided by PPC [27]. Children who received pediatric palliative home care were more likely to have fun (70% versus 45%) and to experience events that added meaning to life (89% versus 63%) [9]. In addition, families who received PPC services report improved communication [28] and children receiving PPC experience shorter hospitalizations and fewer emergency department visits [29]. Parents of children with cancer who received PPC reported less distress from pain, dyspnea and anxiety during the end-of-life period [8]. Undertaking research in PPC is inherently difficult, and Nelson et al. in this special issue address this in their article [30].

The advanced pain and symptom management may explain the increase of survival in patients with serious illness enrolled into PPC. Brock et al. describe in this special edition describe emerging methods of symptom and health-related quality-of-life assessment through patient-reported outcomes tools [31]. Data has shown, that distressing pain is very common among inpatients referred to palliative care and three-quarters of patients with pain improve and improvement in pain is associated with other symptom improvement [32]. The involvement of PPC team with adolescent and young adult oncology patients is associated with the receipt of less intensive treatments during the last month of life, such as being on a ventilator, invasive procedures, and fewer deaths in the intensive care unit [33].

Advanced pain management for children with serious illness often requires multimodal analgesia. This describes an approach of utilizing multiple analgesic agents (such as basic analgesia, opioids, adjuvant analgesia), regional anesthesia (such as nerve blocks or neuroaxial analgesia), rehabilitation (such as physical therapy, motor graded imagery), psychological (such as cognitive behavioral therapy) and integrative (formally known as “non-pharmacological”) therapies (such as massage, hypnosis) which usually act synergistically for more effective pediatric pain control with fewer side effects than a single analgesic or modality [34,35,36,37].

Harlow and Hain [38] explore the concept of total pain in the largest group of children with life-limiting diseases, pediatric patients with severe neurological impairment.

### 3.4. Myth 4: PPC Can Only Be Provided Comprehensively within a Children’s Hospital

Currently most children in resource rich countries die in a hospital, most commonly in an intensive care unit. In a recent study [39] reviewing all pediatric intensive care unit (PICU) admissions over 15 months of 89,127 children in the United Kingdom, children with life-limiting conditions constituted 57.6% of all admissions. Of the 4821 children who died on the PICU during that period, 72.9% had a life-limiting condition. Since most of the sickest children with serious illness are being taken care of in a hospital, every children’s hospital is now expected to offer an interdisciplinary palliative care service as the standard of care [40].

One of the early PPC pioneers, Dr. Ann Goldman from the Department of Hematology/Oncology at Great Ormond Street in London, UK implemented a “Symptom Care Team,” a team of nurses, who were introduced at cancer diagnosis to the child and family. All children received home visits after their first discharge. Children with high-risk cancer or relapses then already knew the “Symptom Care Team”, which provided a 24/7 service, from the time of diagnosis. Between 1978–1981, before the implementation of the “Symptom Care Team” only 19% of children with cancer died at home. Then, between 1989–1990, after implementation of the team 77% of the children dying from malignancies did so at home [41]. 

The death of a child with a serious illness at home may promote better family adjustment and healing [42,43]. This could be related to decrease helplessness and increased family intimacy by being at home. On the other hand, some have reported that family relationships appeared to be better when the child died in the hospital [44]. While it is often suggested that most children prefer to die at home, this has actually not been systematically evaluated.

Parents of terminally ill children often wish for home care [45,46], and there is a not surprisingly a positive correlation between availability of palliative home care and the number of children dying at home [9,47,48,49]. The interdisciplinary PPC Team involvement in compassionate extubation at home has been explored by Postier et al. in this special issue [50]. Most families regard caring for their dying child as a positive experience [45]. It is critically important to discuss preferences regarding the primary location of care as early as possible. A parental decision to care for their terminally ill child at home involves consideration of medical, psychological, social and cultural factors together with such practical considerations as the availability of respite care, physician access, and financial resources. Children with advanced cancer who also received PPC home care or hospice were significantly more likely to have fun (70% versus 45%), experience events that added meaning to life (89% versus 63%), and to die at home (93% versus 20%) [9]. Whatever the decision is regarding the primary location of care, families should be reassured that they can change from one option to another and that the primary team will remain closely involved [45].

There appears to be a growing consensus of most pediatric palliative care specialists, that advanced PPC for children with serious illness needs to be offered and coordinated primarily by an interdisciplinary team within tertiary pediatric hospitals, aided by an outpatient PPC clinic and must include a palliative home care service [51]. When these services are in place (but not instead), a freestanding hospice and respite house represents an excellent addition (but not substitution) of the services provided inpatient, in clinic, and at home. The World Health Organization (WHO) describes that palliative care can be provided in tertiary care facilities, in outpatient clinics, in community health and hospice centers, and in children’s homes [52]. 

A key component of family support, in addition to addressing the needs of the sick child [53], must be geared toward the siblings [54,55] and parents/caregivers [56]. 

### 3.5. Myth 5: Patients and Their Parents Need to Make a Choice between “Fighting for a Cure” or “to Give up Hope”

Even when there might not be a realistic hope for cure, pediatric patients with a serious illness and their parents may opt for continued disease directed treatment [21]. This might be motivated by the desire to extend life, to palliate symptoms related to progressive disease, or the hope for a miracle. In discussions of treatment options with families, one might choose statements, as suggested by Wolfe, such as “The very nature of miracles is that they are rare. However, we have seen miracles, and they have occurred both on and off treatment” [57]. In other words, a child does not have to continue on disease-directed therapy in order to preserve hope, especially when the therapy significantly impacts the child’s remaining quality of life. In fact, a large adult study could demonstrate that chemotherapy for end-stage cancer does not prolong life, however reduces the quality of life [58]. 

Caring for a child at end-of-life is emotionally very difficult. It may be particularly challenging for clinicians and parents to consider the early integration of PPC because this may be perceived as ‘giving up’. As a result, the emotional cost of the recognition that a child may die could impede planning for optimal pain and symptom management and psycho-social-spiritual support. In fact, the “hope for cure” and “pediatric palliative care” include and complement each other. PPC translates into advanced management to maintain or improve quality of life and children can graduate from PPC. No matter the treatment goals, despite the prevailing myth, disease-directed care and excellent symptom relief must and can be provided simultaneously.

Date has shown that the integration of PPC explicitly does not result in giving up hope. Engaging in advanced care planning increases the patients knowledge without diminishing hope, increasing hopelessness, nor inducing anxiety in patients with advanced cancer [59]. The disclosure of a terminal prognosis does not mean loss of patient hope: Instead, hope was redefined on a goal other than cure [60]. In pediatrics, there is no evidence that prognostic disclosure makes parents less hopeful. Instead, the disclosure of a prognosis by physician can support hope, even when prognosis is poor [61]. Parents who are upset by prognostic information are no less likely to want it. The upsetting nature of prognostic information does not diminish parents’ desire for such information, its importance to decision making, or parents’ sense of hope [62]. 

During a goals of care discussion with a family of child with a serious illness, a question such as “Considering what your child is up against, what are you hoping for?” may be posed by the clinician. Not surprisingly, the response may be “Cure from the disease” or “A miracle”. The clinician may then respond “I hope this too. Just in case the disease cannot be cured—What else are you hoping for?” By exploring the extend of hope further, families may wish for very advanced treatment and prevention of pain and distressing symptoms, the possibility to go home, for grandmother to visit, to hold their child more often, or many other things. Blazin et al. in this special edition explore how to translate evidence into practice and communicate effectively [30]. Even when the underlying condition cannot be cured, PPC will never give up hope. Sometimes, it appears that the best chance for these children to truly live is for their parents and treating clinicians to accept the fact that this child might die [63]. Not surprisingly, data reveals, that religion, spirituality or life-philosophy play an important role in the life of most parents whose children receiving PPC [64].

### 3.6. Myth 6: Administering Morphine Causes Respiratory Depression and Hastens Death

To paraphrase Sykes [65] “Morphine kills the pain, not the patient. A physician killing a patient in name of pain or dyspnea relief is not merciful, just incompetent.” An enduring misconception is the belief that in the management of pain and dyspnea, opioids will hasten death and should only be administered as a last resort. This was contradicted in the adult literature [66] and our PPC teams commonly observe that administering opioids and/or benzodiazepines, together with comfort care to relieve dyspnea and pain, not only improves the child’s quality of life, but also prolongs life [67]. A retrospective cohort study (n = 223 adult oncologic patients) reviewing the mean survival in relation to opioid use found less than a two-fold increase in their initial opioid dose resulted in 9 days survival, and more than a two-fold increase in 22 days survival [68]. 

One of the most common sources of pain and distress in children is visceral hyperalgesia and feeding intolerance, addressed by Hauer in this Special Issue [69].

### 3.7. Myth 7: PPC Teams Are Too Expensive 

A pediatric palliative care team is now expected standard of practice in every children’s hospital. Every single one of the “U.S. News & World Report honor roll children’s hospitals” have palliative care teams [70]. Overwhelming evidence now demonstrates, that children’s hospitals in fact cannot afford to *not* have a PPC team anymore. PPC team basically “grease the wheels” of a clinical institution, such as reducing burnout and as a result staff turn-over among pediatric staff. For instance, in the United States more than 30% of all new nurses elect either to change positions or leaving nursing completely within the first 3 years of clinical practice [71]. Leading reasons include emotional distress related to patient care. The average cost of turnover for a bedside nurse ranges from $36,900 to $57,300, resulting in the average hospital losing $4.9 million–$7.6 million per year. Each percent reduction in nursing turnover will cost/safe the average hospital an additional $379,500 [72]. PPC services can help reducing turnover and improving overall job satisfaction and performance important in to assuring patient and family satisfaction while promoting quality care.

In addition to providing advanced clinical care, pediatric palliative care services are also cost-effective (although arguably not a single PPC team member worldwide provides clinical care “to safe money” to the institutions or health insurers, but rather, because it provides superior clinical care to children with advanced serious illnesses. PPC involvement results in better outcomes and lower costs: Palliative care program reduces stress, costs of care for children with life-threatening conditions [73,74,75]. A retrospective study of 425 children receiving pediatric palliative home care in the US comparing pediatric hospital resource utilization before and after PPC enrollment decreased in non-cancer patients the length-of-hospital-stay by 38 days and decreased hospital charges $ 275,000 per patient [76]. 

## 4. Conclusions

Despite significant growth and advances over the last two decades, a large number of children’s hospitals currently either do not have a PPC service, or it significantly under-resourced and underfunded. When implementing a PPC service, the development often goes through four steps in their development. These should be anticipated, and an action plan might include

- **Denial**: Document unmet needs, undertake surveys among staff, patients and providers
- **Palliphobia**: Close collaboration with colleagues; disciplined planning, rapid conflict resolution
- **Pallilalia**: Documentation of PPC value to leadership; grand rounds by expert in field and external review
- **Palliactive**: “Do good & talk about it”, perform QI, evaluate value of PPC in ACO/bundled payments environment; include clinical and administrative innovators in program development
- **Secure funding:** “Make sure your passion is connected with somebody’s payment system”

High-quality pediatric palliative care for children with serious illnesses is now an expected standard of medicine. However, even in resource-rich settings, there remain significant barriers to achieving optimal care related to lack of formal education, reimbursement issues, the emotional impact of caring for a dying child, and most importantly the lack of interdisciplinary PPC teams with sufficient staffing. Whenever possible, treatment should focus on continued efforts to control the underlying illness. At the same time, children and their families should have access to interdisciplinary care aimed at promoting optimal physical, psychological and spiritual wellbeing. Persistent myths and misconceptions have led to inadequate symptom control in children with life-limiting diseases. Pediatric Palliative Care advocates the provision of comfort care, pain, and symptom management concurrently with disease-directed treatments. Families no longer have to opt for one. They can pursue both, and include integrative care to maximize the child’s quality of life.
